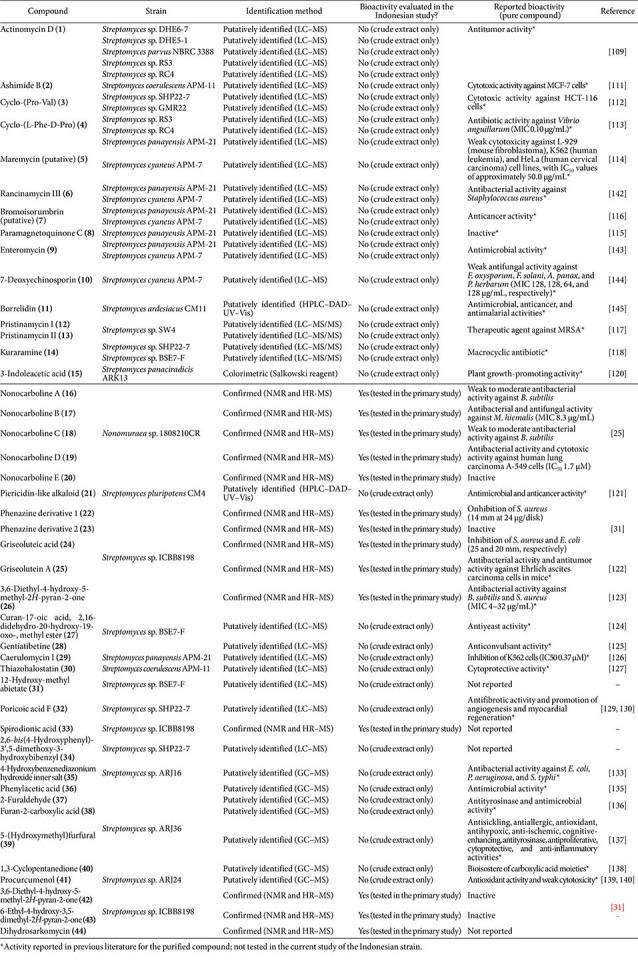# Biodiversity and Natural Product Potential of Indonesian Actinomycetes

**DOI:** 10.4014/jmb.2603.03051

**Published:** 2026-07-29

**Authors:** Erin Karissa, Widya Esti Purwaningtyas, Ganiyu Akintayo Akinniyi, Hiyoung Kim, Inho Yang

**Affiliations:** 1 Department of Marine Life Sciences and Environment, Korea Maritime and Ocean University, Busan 49112, Republic of Korea; 2 Department of Biology, Faculty of Mathematics and Natural Science, Udayana University, Bali 80361, Indonesia; 3 Department of Convergence Study on the Ocean Science and Technology, Korea Maritime and Ocean University, Busan 49112, Republic of Korea; 4 Department of Biomedical Science & Engineering, Konkuk University, Seoul 05029, Republic of Korea

**Keywords:** Actinomycetes, Biodiversity, Indonesia, Phylogenetic analysis, Microbial natural product

## Abstract

Actinomycetes are a diverse group of microorganisms that play essential roles in environmental processes and drug discovery, particularly through the production of bioactive secondary metabolites. In Indonesia, most actinomycetes have been isolated predominantly from soil; however, the country encompasses a wide range of underexplored ecosystems, including karst ecosystems, geothermal areas, mangroves, marine organisms, and plant-associated environments. This review analyzes the biodiversity of actinomycetes across different environments in this island nation by constructing a phylogenetic tree based on 152 strains with available 16S rRNA gene sequences. This analysis revealed a broad distribution of actinomycete taxa, with the majority belonging to the genus *Streptomyces*. A total of 44 actinomycete-derived natural products, comprising both structurally elucidated and putatively identified compounds, have been reported in previous studies. These findings indicate that natural products from Indonesian actinomycetes remain underexplored compared with global reports. Furthermore, this review identified that molecular-level identification remains limited and suggests future studies to increase DNA sequencing efforts and deposit the resulting data in public databases to enhance taxonomic precision and facilitate future research in natural product discovery and phylogenetics.

## Introduction

Indonesia, one of the world's largest archipelagic nations, comprises more than 17,500 islands and has a coastline extending over 80,000 km. This geographical vastness supports a wide array of coastal and marine ecosystems, including beaches, dunes, estuaries, mangroves, coral reefs, seagrass beds, tidal forests, coastal mudflats, algal beds, and various small island ecosystems [1].

This richness in biodiversity is also reflected in its vast and underexplored microbial diversity. Despite this potential, efforts to isolate and characterize microorganisms remain limited and require greater attention [2]. Microbial biodiversity and its genetic potential in Indonesia have been insufficiently studied compared with plants and animals [3]. Among these microorganisms, actinomycetes have attracted particular attention because of their filamentous structure, which resembles that of both bacteria and fungi, as well as their crucial roles in ecological processes and biotechnological applications [4].

Several studies have reported the presence and bioactivities of actinomycetes across Southeast Asia. In 2023, a study that collected oil-contaminated soil from the northern states of Malaysia yielded *Streptomyces* sp. RP1, which demonstrated bioremediation capabilities. This microorganism produced lipase and citrate, key metabolites involved in hydrocarbon degradation. The strain also exhibited high emulsification activity against kerosene, engine oil, and an olive oil–diesel mixture, with a substantial reduction in surface tension [5]. Another study from Thailand reported that *Streptomyces* sp. S2-SC16, isolated from a mangrove ecosystem, exhibited strong inhibitory activity against the fungus *Fusarium fujikuroi* CMU-F02 and promoted rice seedling growth by significantly enhancing several growth parameters, including root length, shoot height, leaf width, and biomass [6].

In Indonesia, actinomycetes have been isolated from a wide range of unique environments, including soil, marine organisms, geothermal areas, plants, marine sediments, and karst ecosystems. Novel actinomycete strains have been reported, including *Actinokineospora* spp. isolated from soil and plant litter in Cianjur and Bali [7], and *Rhodococcus indonesiensis* isolated from the sediment of a neutral hot spring in Sukabumi [8]. Additionally, several strains have been reported to produce promising natural compounds. For example, *Streptomyces* sp. RS3 and *Streptomyces* sp. RC4, isolated from a marine sponge and coral, respectively, were found to produce actinomycin D with antibacterial activity [9]. These findings highlight Indonesia’s significant potential as a valuable reservoir of actinomycete biodiversity and natural product discovery.

Although research on actinomycetes in Indonesia has increased in recent years, detailed information on their biodiversity, molecular sequences, and secondary metabolite–producing capacity remains limited. Therefore, this review provides a systematic overview of actinomycete diversity in Indonesia based on the phylogenetic relationships of strains isolated from various environments, alongside the natural products and bioactivities reported in the literature. In addition, this review identifies knowledge gaps and research needs to guide future investigations and support the advancement of actinomycete studies in Indonesia.

## Methodology

### Search Strategy

**Literature search strategy.** This systematic review was conducted to identify studies on actinomycetes in Indonesia. The literature search was performed across two major databases: Google Scholar (in both English and Indonesian) and ScienceDirect. This review included relevant studies on actinomycetes in Indonesia, from the earliest identified record in 2008 through March 2025. To maintain thematic precision, the search was limited to the first 10–15 pages of results from each database. This decision was based on a preliminary assessment that showed a significant decline in relevance beyond the initial pages. The specific search strings used for each database are detailed in Table 1.

### Publication Screening

**Fig. 1:**
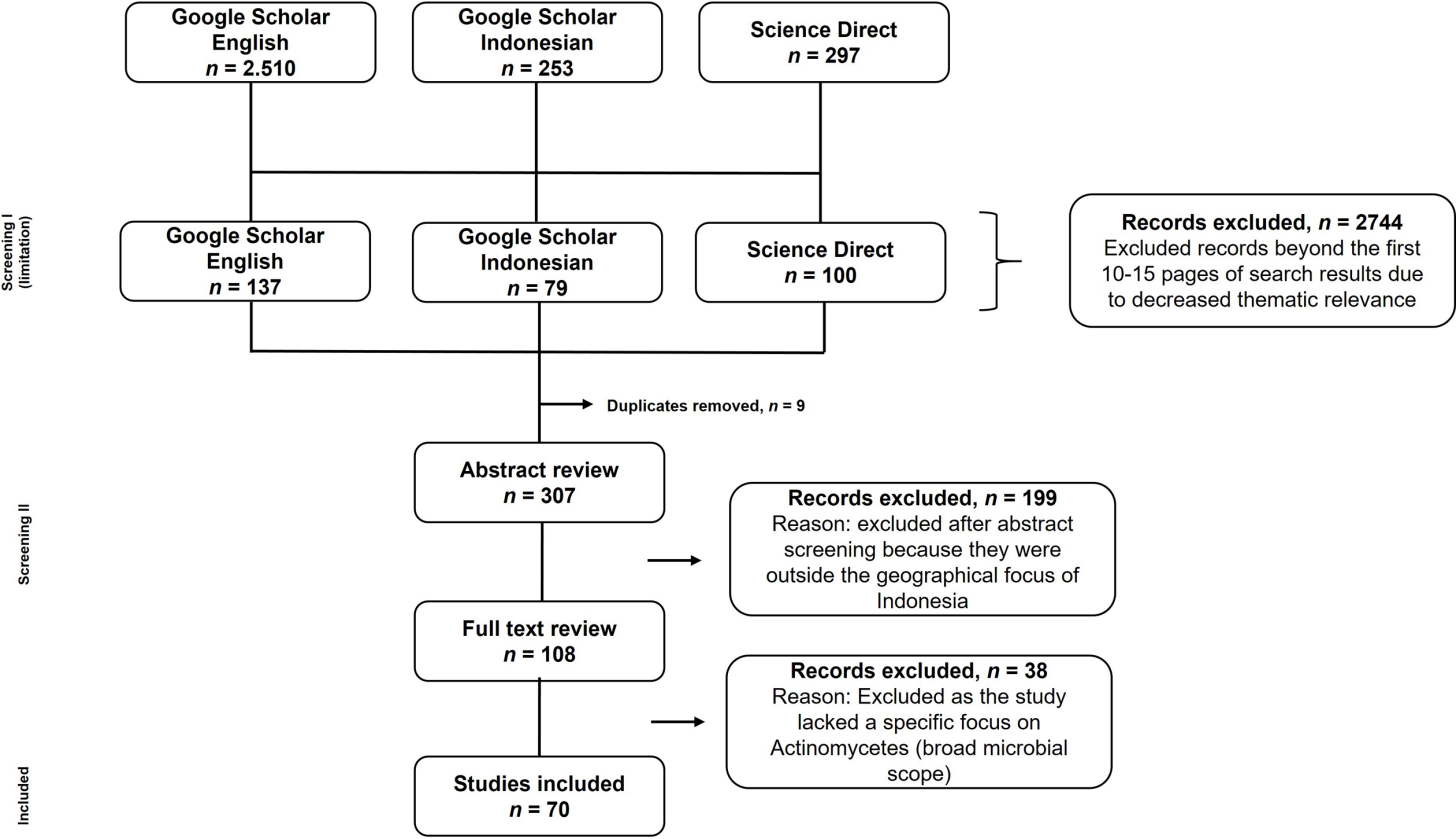
PRISMA flow diagram for the literature search and publication screening process.

Fig. 1 presents the PRISMA flow diagram detailing the publication screening process. An initial search across two scientific databases—Google Scholar (English and Indonesian) and ScienceDirect—identified 3,060 records based on the systematic search strings provided in Table 1. Page limitation were applied to balance comprehensiveness with screening efficiency. The search was discontinued after the first 10–15 pages based on a data saturation stopping rule: we stopped screening if three consecutive pages turned up nothing but duplicates or irrelevant studies (*e.g.*, broad microbial ecology papers). Consequently, 316 records were retrieved for further screening.

Reference management and duplicate identification were performed using Mendeley Reference Manager, and 9 duplicates were removed. The remaining 307 abstracts were screened, and 199 were excluded because they were outside the geographical scope of Indonesia. In the final screening stage, 108 full-text articles were reviewed, and 38 were excluded because of insufficient taxonomic specificity (*i.e.*, studies with a broad microbial scope). Ultimately, 70 papers met all eligibility criteria and were included in this review.

### Data Collection Process

All strain-related data included in this review were extracted from published research articles and organized according to their isolation locations. For genetic analyses, 16S rRNA gene sequences were retrieved using the accession numbers provided in the articles and downloaded from public databases, such as the National Center for Biotechnology Information (NCBI) (https://www.ncbi.nlm.nih.gov/). Phylogenetic analysis was conducted using Geneious Prime software (version 2025.0.3) to construct the phylogenetic tree. Secondary metabolites reported for each strain were compiled and categorized according to their chemical structures, which were drawn using ChemSketch software (version 2024.2.3).

### Sources of Actinomycetes in Indonesia

**Fig. 2:**
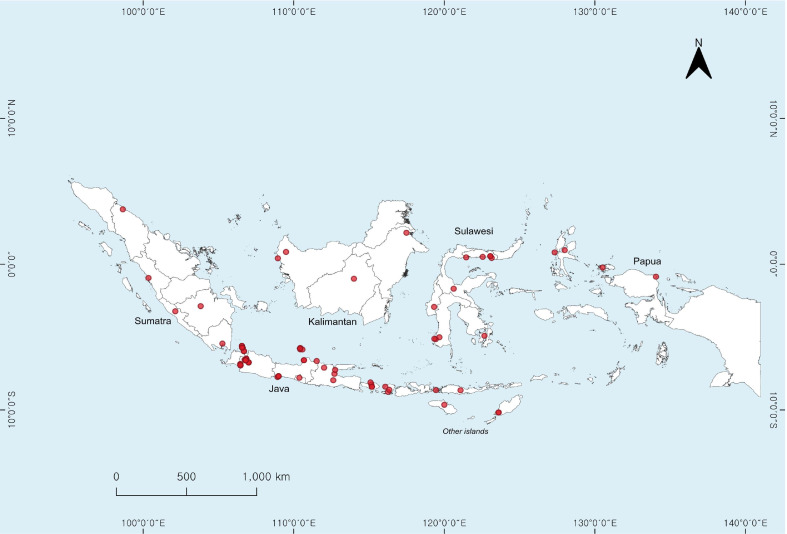
Distribution of actinomycetes study sites in indonesia.

Actinomycetes in Indonesia have been isolated from a wide range of environments. In this review, these isolation sources were grouped into six main categories: soil, karst ecosystems, geothermal areas, mangrove ecosystems, marine organisms, and plant-associated environments. Based on the published articles, we identified 78 study sites across the nation. These sites were further categorized according to the five major islands—Sumatra, Java, Kalimantan, Sulawesi, and Papua—arranged from west to east. Other islands, including Bali, Nusa Tenggara, Maluku, and surrounding archipelagos, were grouped separately as “other islands” due to their fragmented geography. We found that previous studies are unevenly distributed across Indonesia, with a heavy concentration of research conducted on Java Island (42 study sites), followed by the other islands (14 study sites), Sulawesi (11 study sites), Sumatra (5 study sites), Kalimantan (4 study sites), and Papua (2 study sites). However, since most of the papers did not include exact GPS coordinates, these study sites are represented by their approximate or central geographic locations, as visualized in Fig. 2.

### Soil Ecosystems

Actinomycetes are widely distributed in soil, and their abundance is strongly influenced by environmental factors such as temperature, soil type, pH, organic matter content, cultivation practices, aeration, and moisture content [10]. Indonesia exhibits substantial variation in soil characteristics because of differences in geomorphology, volcanic activity, and climate, thereby creating environmentally distinct habitats that may drive microbial diversification. For instance, soils in Sumatra are generally less fertile, whereas Java and Bali benefit from nutrient-rich volcanic deposits derived from active volcanoes such as Mount Merapi. Kalimantan is largely dominated by alluvial soils that are relatively infertile, while soils in Sulawesi often contain high magnesium concentrations and heavy metals resulting from geomorphological and volcanic processes. In Nusa Tenggara, soil fertility varies geographically, with more productive northern regions and drier, less fertile southern areas [11].

This heterogeneity creates a broad range of ecological niches that may support diverse and potentially unique actinomycete communities. Because actinomycete diversity is closely associated with the chemical, physical, and biological characteristics of their environment, identifying unconventional habitats is crucial for the discovery of novel species and their associated bioactive metabolites [12].

Several studies have reported the successful isolation of actinomycetes from various soil types in Indonesia, including agricultural lands. Rice field soils, for example, have been recognized as important reservoirs of actinomycetes. One study isolated 24 strains from rice field soils in Central Lombok Island, representing five genera—*Dermacoccus*, *Actinoplanes*, *Micromonospora*, *Pseudonocardia*, and *Streptomyces*—based on morphological identification [13]. Another study from rice fields in Bogor, Java, identified 17 strains belonging to the genera *Geodermatophilus*, *Actinokineospora*, *Actinoplanes*, *Streptomyces*, and *Kocuria* [14].

Furthermore, potentially novel actinomycete species have been discovered in different soils in Indonesia. A potentially novel *Streptomyces* species was reported from garbage dump soil in Surabaya and was represented by several strains, including Sp-D, Sp-Ep, Sp-G, and Sp-I [15]. Overall, soil-derived actinomycetes from Indonesia represent a valuable resource for the discovery of novel actinomycete species. To illustrate the diversity of isolated actinomycete species, selected findings from various Indonesian regions are summarized in Table 2.

### Karst Ecosystem

Karst refers to a unique landscape characterized by caves and soluble rocks, such as limestone, marble, and gypsum, resulting in extensive underground water systems. Globally, an estimated 20–25% of the population relies on groundwater originating from karst ecosystems for daily use [42]. Karst environments exhibit distinct features, including soluble rock formations, alkaline and calcium-rich substrates, limited soil availability, a double-layer soil structure, high porosity, and dark subterranean spaces with relatively stable temperatures and elevated humidity [43]. Organisms inhabiting karst ecosystems must adapt to high alkalinity, thin soil layers, and moisture loss occurring on porous limestone surfaces. Moreover, because of low light availability and limited water, organic matter, and variable gas concentrations within caves, organisms require substantial physiological adaptations for survival [44]. Despite these extreme conditions, actinomycetes have been reported as dominant members of karst microbial communities [45].

Actinomycetes isolated from Indonesian karst environments have demonstrated notable diversity. For example, *Streptomyces* sp. KSLI and *Streptomyces* sp. KSIc, obtained from the Bangga Mountain, Olohuta Hills, and Panipi Hills karst systems in Gorontalo, exhibited biological activity in a 2025 study [46]. Similarly, research conducted in Bantimurung–Bulusaraung National Park, South Sulawesi, reported eight isolates, of which two—*Streptomyces* sp. B.11 and *Streptomyces* sp. B.17—showed antifungal potential [47].

Although karst ecosystems present harsh and highly specialized environmental conditions, they represent a promising source of actinomycetes with unique adaptations that may be associated with the production of novel metabolites. A summary of actinomycetes isolated from Indonesian karst environments is presented in Table 3.

### Geothermal Area

Geothermal areas are among the rare environments that provide valuable insights into early Earth life and evolution [50]. Indonesia, located between the Ring of Fire and the Alpide Belt, experiences intense volcanic activity. As of 2023, the Ministry of Energy and Mineral Resources reported 222 geothermal greenfields and 62 geothermal working areas, making Indonesia one of the world’s most geothermally active regions. These environments support rich microbial diversity, particularly thermophilic prokaryotes that thrive at high temperatures [51]. Thermophilic actinomycetes, often found in thermal ecosystems, can survive at elevated temperatures ranging from 40°C to 80°C. Their survival is largely attributed to adaptive mechanisms, such as enhanced stability of cellular components and the production of heat-resistant enzymes [52]. These unique physiological adaptations make them promising sources of secondary metabolites with biotechnological potential.

A recent study in 2024 reported a new member of the *Rhodococcus ruber* lineage, *Rhodococcus indonesiensis* sp. nov., isolated from non-saline, neutral hot spring sediments of the Cilosok geyser in Sukabumi, West Java Province. The strain grows at 10–37°C and tolerates up to 10% NaCl, forms reddish-orange colonies, and produces various hydrolytic enzymes, highlighting its biotechnological potential [53]. Another rare actinomycete isolated from soil samples collected under bamboo trees in the same Cilosok geothermal area showed the closest phylogenetic relationship to *Dictyobacter aurantiacus* S-27^T^ within the class Ktedonobacteria [54]. Given Indonesia's extensive geothermal regions, there is significant potential to isolate rare microorganisms and explore their capacity as sources of novel bioactive compounds. To illustrate the diversity of isolated genera and their reported bioactivities, selected findings from various Indonesian geothermal regions are summarized in Table 4.

### Mangrove Ecosystem

Mangrove forests are often referred to as “coastal woodlands” or “tidal forests” [57]. The term mangrove tree refers to woody shrubs, palms, and trees that inhabit tidal wetlands along tropical and subtropical coastlines [58]. These environments are strongly influenced by fluctuating sea tides, and mangrove plants have evolved remarkable adaptations to cope with high salinity and anaerobic soil conditions. Mangroves adapt through specialized root structures, such as pneumatophores, knee roots, stilt roots, buttress roots, and aerial roots, which facilitate oxygen uptake in anaerobic soils [59]. They also exhibit salt tolerance through mechanisms including ion regulation, isolation of salt from sensitive organelles, storage of Na+ and Cl− ions, synthesis of osmotically compatible solutes, and, in some species, salt excretion through salt glands [60]. Furthermore, mangrove ecosystems host diverse microbial communities because of their high organic carbon content, which supports active carbon and nutrient cycling [61].

A study conducted in 2021 reported the isolation of two *Streptomyces* strains, *Streptomyces* sp. BSE7F and *Streptomyces* sp. SHP22-7, from mangrove sediment on Enggano Island, North Bengkulu, Sumatra [30]. Given Indonesia’s extensive mangrove coverage, these ecosystems represent a promising source for the discovery of ecologically distinct actinomycetes. Selected reports of actinomycete genera isolated from Indonesian mangrove habitats are summarized in Table 5.

### Marine Organisms

As the rate of discovery of novel bioactive compounds from terrestrial actinomycetes declines, attention has increasingly shifted toward marine environments as a promising alternative source [71]. Marine environments possess unique ecological conditions that differ markedly from those of terrestrial habitats, potentially driving microorganisms to produce structurally distinct secondary metabolites [72]. In marine environments, actinomycetes are commonly isolated from sediments, seawater, and marine organisms, particularly sponges, ascidians, corals, and brown algae [73].

In Indonesia, actinomycetes have been successfully isolated from various marine invertebrates. For instance, *Streptomyces* sp. RS3 and *Streptomyces* sp. RC4 were isolated from corals and sponges collected from Baru, Randayan, and Lemukutan Islands in Bengkayang District, West Kalimantan [9]. In another study, nudibranch-associated actinomycetes obtained from the coastal waters of Bandengan and Panjang Island, Jepara, Central Java, yielded 27 isolates from *Jorunna* sp. and 12 from *Chromodoris* sp. Among these isolates, *Streptomyces* sp. NPC8 demonstrated strong inhibitory activity against multidrug-resistant (MDR) pathogens, highlighting the pharmaceutical potential of invertebrate-associated actinomycetes [74]. More recently, surveys of seagrass-associated bacteria in the Karimunjawa Sea and Awur Bay recovered 70 actinomycete isolates (41 endophytes and 29 epiphytes) from various seagrass species, several of which exhibited activity against methicillin-resistant *Staphylococcus aureus* (MRSA) and other MDR pathogens [75]. Together, these studies highlight the remarkable diversity of marine organism–associated actinomycetes in Indonesia. A summary of actinomycete genera isolated from marine organisms in Indonesia is presented in Table 6.

### Plant-Associated

Actinomycetes are widely recognized as endophytic microorganisms that colonize the internal tissues of host plants, often forming mutualistic associations and contributing to host defense through the production of bioactive metabolites [85]. Within host plants, actinomycetes play important roles, particularly in the roots, where they contribute to the biocontrol of pathogens [86] oxidize dead plant material to produce nitrogenous and mineral compounds, and decompose organic matter [87]. By breaking down complex plant materials, actinomycetes enhance the availability of nitrogenous and mineral compounds essential for plant growth and participate in humus formation, which gives soil its characteristic dark color.

In Indonesia, several studies have explored the diversity of plant-associated actinomycetes. In 2021, six *Streptomyces* and two *Microbispora* strains were isolated from the rhizome and stem of the herb *Curcuma zedoaria* in Bojonggede, Bogor. These isolates showed promising antifungal and broad-spectrum antibacterial activities and exhibited substantial potential for development as sources of new bioactive compounds [18]. Another study on endophytic actinomycetes isolated from medicinal plants maintained at the Collection of Medicinal Plants Garden of the Tropical Biopharmaca Research Center, Bogor Agricultural University, Bogor, successfully isolated 65 endophytic actinomycetes. One isolate, *Streptomyces* sp. BWA65, displayed 92% 16S rRNA gene sequence similarity to *Streptomyces olivochromogenes*, which had been isolated from the flowering plant *Tinospora crispa*. This low sequence similarity suggests that the strain may represent a novel species [88]. Actinomycetes residing within host plants not only provide benefits to the host but also play a key role in the production of valuable natural compounds. These findings underscore the ecological significance of plant-associated actinomycetes in Indonesia. To illustrate the diversity of isolated genera, selected findings from various Indonesian regions are summarized in Table 7.

### Phylogenetic Analysis of Actinomycetes Isolated from Indonesia

Although 16S rRNA gene sequencing remains the most widely used method for actinomycete identification in Indonesia, its application is still constrained by high operational costs. Consequently, many studies rely primarily on morphological characterization, with only selected or potentially bioactive strains subjected to further molecular identification [13]. However, morphological approaches are generally labor-intensive, time-consuming, and limited in taxonomic resolution [96]. Therefore, 16S rRNA gene sequencing plays a crucial role in species-level identification and in interpreting phylogenetic relationships among strains [97].

A strain is generally considered a potential novel species when its 16S rRNA gene sequence similarity is below the threshold of 98.65% compared with its closest relatives [98]. In a study published in 2016, the identification of 229 actinomycete isolates based on 16S rRNA gene sequencing revealed that approximately 58 isolates (25%) exhibited less than 98% sequence similarity, indicating the potential presence of novel species or even novel genera that require further taxonomic investigation [36]. Despite several studies reporting 16S rRNA gene sequences of actinomycetes from diverse environments in Indonesia, the number of formally described novel species remains relatively low. This review compiles the available 16S rRNA gene sequences of Indonesian actinomycetes and examines them within a unified phylogenetic framework to improve understanding of strain diversity across different ecological habitats.

The sequence data were aligned using the MUSCLE v5.1 algorithm [99]. Phylogenetic trees were constructed using the Maximum Likelihood (ML) [100] method in MEGA 11 to ensure robust evolutionary inference. The Tamura–Nei [101] model was applied as the nucleotide substitution model with uniform rates among sites. To handle gaps and missing data, a partial deletion strategy with a 95% site coverage cutoff was implemented.

**Fig. 3:**
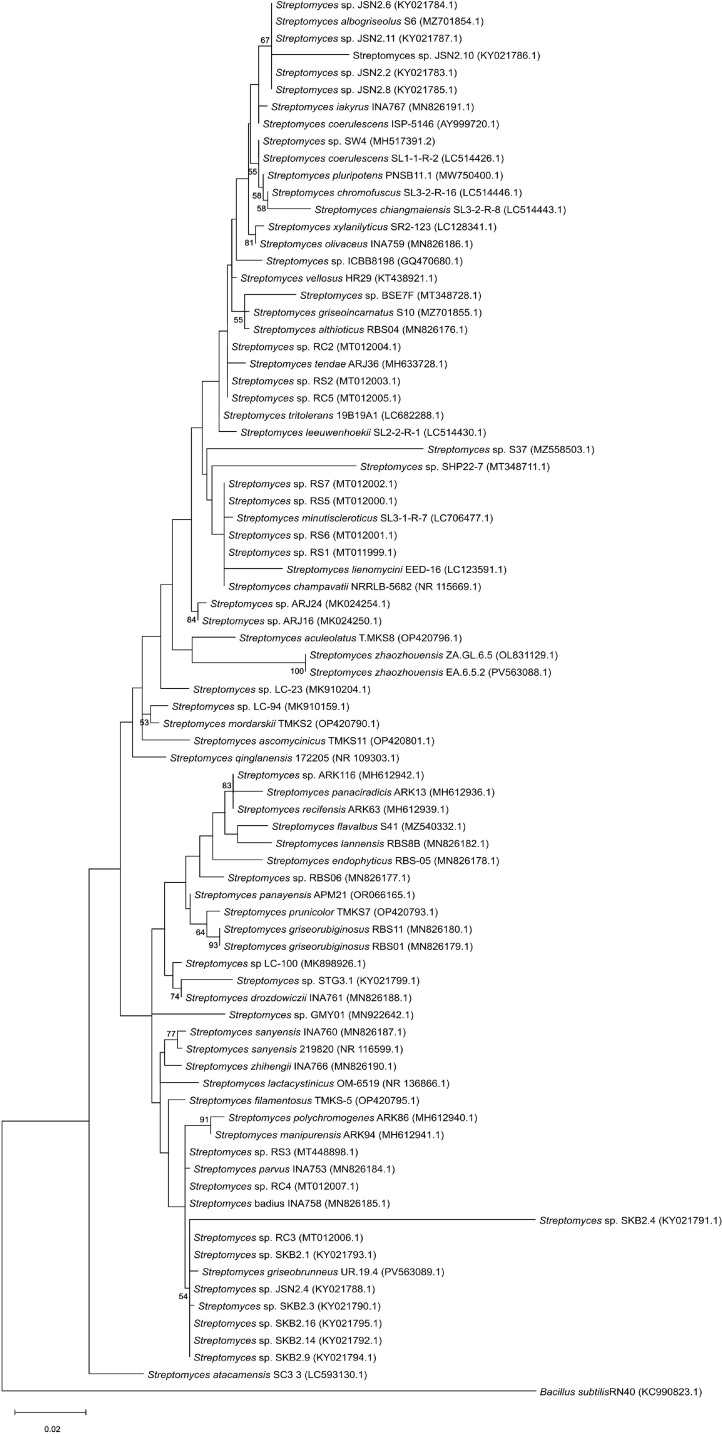
Phylogenetic tree of Indonesian actinomycetes belonging to the genus *Streptomyces*. The numbers at the branch nodes represent bootstrap support values based on 1,000 resamplings; only values ≥ 50% are shown.

**Fig. 4:**
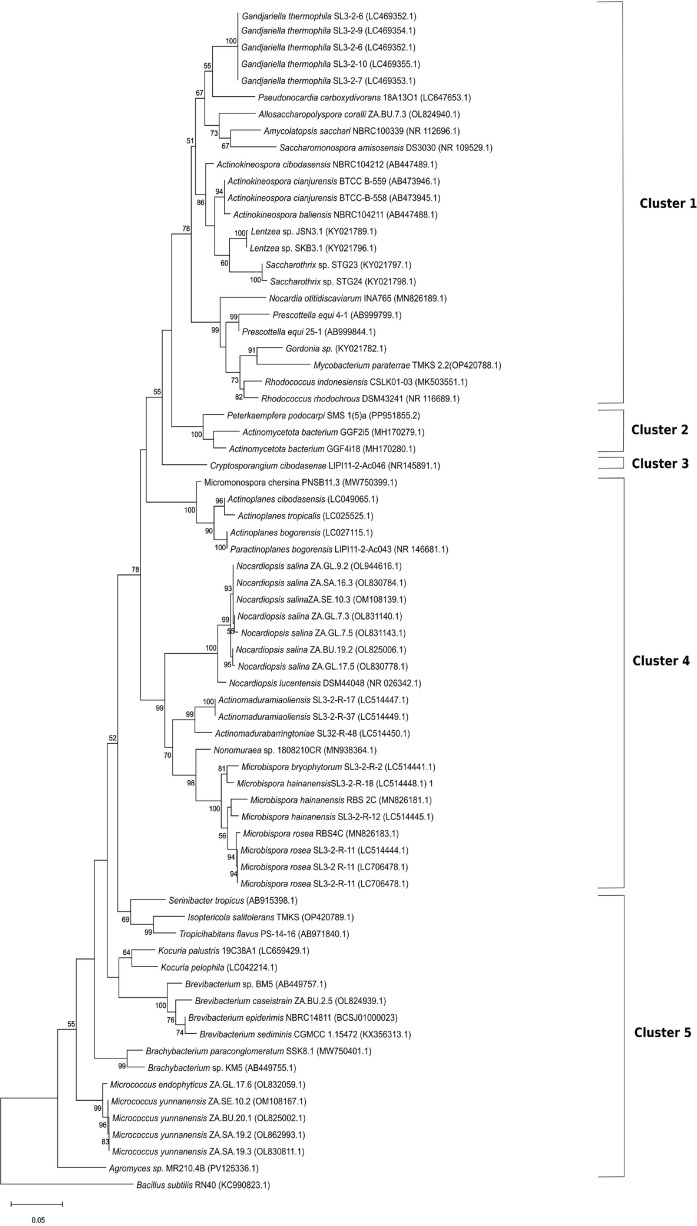
Phylogenetic tree of Indonesian non-Streptomyces (rare) actinomycetes. The numbers at the branch nodes represent bootstrap support values based on 1,000 resamplings; only values ≥ 50% are shown.

The reliability of the tree topologies was evaluated using bootstrap analysis with 1,000 resamplings. For the genus *Streptomyces*, the final alignment consensus length was 1,485 bp, whereas for the non-*Streptomyces* group (rare actinomycetes), the alignment length was 1,360 bp. *Bacillus subtilis* RN40 was used as the designated outgroup to provide a clear evolutionary root. To optimize visual readability, the phylogenetic analysis was presented in separate figures for each group. The phylogenetic trees of the genus *Streptomyces* are presented in Fig. 3, and those of the non-*Streptomyces* group (rare actinomycetes) are presented in Fig. 4.

Based on the phylogenetic tree, actinomycetes in Indonesia belonged to two distinct groups, *Streptomyces* and non-*Streptomyces*, with the genus *Streptomyces* representing the largest proportion. Despite being collected from diverse habitats, the isolates exhibited relatively limited phylogenetic diversity, as the majority clustered within the genus *Streptomyces*. This genus comprised 81 strains, accounting for 53% of the currently available genetic data on actinomycetes in Indonesia. Notably, *Streptomyces* sp. SKB2.4, *Streptomyces* sp. STG3.1, and *Streptomyces* sp. SKB2.1 showed low 16S rRNA gene sequence identities of 92%, 95%, and 96%, respectively, compared with their closest type strains, *Streptomyces cavourensis*, *Streptomyces drozdowiczii* PhyCEm-1349, and *Streptomyces cavourensis* A15 [92]. Similarly, *Streptomyces* sp. SHP22-7, isolated from mangrove sediment, showed a low sequence identity of 94.84% compared with its closest type strain, *Streptomyces rochei* NRRL B-1559^T^ [30].

The term “*rare* actinomycetes” refers to non-*Streptomyces* actinomycetes, which are usually isolated at lower frequencies [102]. Indonesian rare actinomycetes spanned six distinct phylogenetic clusters across six orders. Among these, Cluster 1 (order Actinomycetales, based on the Genome Taxonomy Database [GTDB] framework) displayed the highest diversity among rare actinomycetes, comprising four families (*Pseudonocardia*ceae, *Nocardia*ceae, *Gordonia*ceae, and Mycobacteriaceae) and thirteen distinct genera: *Lentzea*, *Saccharothrix*, *Actinokineospora*, *Pseudonocardia*, *Ganjariella*, *Allosaccharopolyspora*, *Amycolatopsis*, *Saccharomonospora*, *Nocardia*, *Prescotella*, *Rhodococcus*, *Gordonia*, and *Mycobacterium*. Notably, most of these isolates originated from marine-associated environments, including mollusks, corals, and marine sediments, underscoring the ecological significance of marine habitats as reservoirs of diverse actinomycetes.

Cluster 2 (order Kitasatosporales) resolved into a group containing the genus *Peterkaempfera*. Notably, *Peterkaempfera podocarpi* SMS1(a)5 represents the most recently described actinomycete species, isolated in 2024 from the rhizosphere of the conifer *Podocarpus rumphii* on Sumba Island, Indonesia [39]. This cluster also included two uncultured Actinomycetota bacteria, with these uncultured strains were supported by a strong bootstrap value of 100%. The discovery of these strains highlights the microbiological distinctiveness of Indonesian environments [103].

Cluster 3 (order Cryptosporangiales) included another rare actinomycete, *Cryptosporangium cibodasense*, which was isolated from leaf litter in Bogor, West Java. According to Bergey’s Manual of Systematic Bacteriology (2018 ed.) [104], this genus is classified under the order Frankiales, along with seven other representative species. However, recent genome-based taxonomic frameworks, such as the GTDB, have proposed a separate order, Cryptosporangiales, to accommodate its distinct phylogenetic lineage.

Cluster 4 (order Micromonosporales) belonged to the family Micromonosporaceae, comprising three genera: *Micromonospora*, *Actinoplanes*, and *Paractinoplanes*. Among these, all isolates from the genus *Actinoplanes* have been reported as novel species [23, 27], reflecting the considerable potential of Indonesian actinomycetes.

Cluster 5 (order Streptosporangiales) comprised three families (Streptosporangiaceae, Thermomonosporaceae, and Nocardiopsaceae) and four genera: *Nonomuraea*, *Microbispora*, *Actinomadura*, and *Nocardiopsis*. Although *Nocardiopsis* is sometimes classified under a separate order, the phylogenetic tree indicated that it clusters closely with members of Streptosporangiales. Therefore, *Nocardiopsis* can still be considered a close relative of *Actinomadura*, *Nonomuraea*, and *Microbispora* [105]. Interestingly, several genera within this order, particularly *Actinomadura* and *Nonomuraea*, have previously been reported as endophytes in terrestrial plants. In the Indonesian context, these genera have predominantly been isolated from plant tissues collected in the Bogor region, West Java, indicating their strong association with terrestrial plant hosts and highlighting the rich biodiversity of tropical environments.

Cluster 6 (order Micrococcales) has also been recovered from various environments and comprises six different families (Promicromonosporaceae, Cellulomonadaceae, Brevibacteriaceae, Dermabacteraceae, Micrococcaceae, and Microbacteriaceae) and eight genera. The genera identified within this order include *Isoptericola*, *Tropicihabitans*, *Brevibacterium*, *Brachybacterium*, *Micrococcus*, *Kocuria*, *Agromyces*, and *Serinibacter*. Among them, a novel actinomycete, *Tropicihabitans flavus* [29] was isolated from the mangrove *Rhizophora mucronata* on Pramuka Island, DKI Jakarta, Indonesia. This strain is characterized by circular, smooth, pale yellow colonies.

Overall, the phylogenetic tree revealed several actinomycete orders, indicating a moderate level of phylogenetic diversity. However, diversity at the genus level appears limited, as the isolates were predominantly affiliated with *Streptomyces*. A similar pattern has been reported in neighboring countries such as Malaysia, where isolation has been conducted mainly in mangrove environments, yet the recovered isolates were largely dominated by *Streptomyces* [106]. Although *Streptomyces* dominates isolate discovery, phylogenetically novel *Streptomyces* species have been more frequently reported from marine environments. In addition, rare actinomycetes have been more frequently reported from extreme or non-soil habitats, including marine, mangrove, and plant-associated environments. These observations suggest that broader exploration of diverse and underexplored ecosystems is necessary to reduce redundancy and avoid repeated rediscovery in actinomycete research in Indonesia.

Although numerous studies on actinomycetes have been conducted in Indonesia, only a limited number provide 16S rRNA gene sequences with valid accession numbers. Consequently, the phylogenetic analysis in this review was restricted to 152 strains compiled from the open-source database NCBI. Therefore, the observed phylogenetic diversity may not fully represent the true actinomycete diversity present in Indonesian environments. Future studies incorporating full-length 16S rRNA gene sequences are essential to more accurately resolve actinomycete diversity in this country.

### Secondary Metabolites

Actinomycetes produce secondary metabolites with significant clinical relevance, contributing up to 45% of bioactive microbial compounds [107]. Unlike primary metabolites, these compounds are not essential for normal growth, but they play important roles in survival and interspecies competition [108]. In this review, the reported secondary metabolites from Indonesian actinomycetes within the specified period were documented and categorized according to their chemical structures.

### Cyclic Peptides

Among the cyclic peptides reported from Indonesian actinomycetes, actinomycin D (**1**) is one of the most frequently encountered metabolites. Compound **1** has been extensively studied and is clinically applied, particularly for its potent antitumor activity [109]. In the context of Indonesian biodiversity, this cyclic nonribosomal peptide has been putatively identified via LC–MS analysis of crude extracts from five different *Streptomyces* strains obtained from both terrestrial and marine environments. For instance, *Streptomyces* sp. DHE6-7 and *Streptomyces* sp. DHE5-1, isolated from the rhizosphere of *Salacca* sp. on Enggano Island, exhibited metabolic profiles consistent with actinomycin D production [110]. While *Streptomyces* sp. DHE6-7 showed antimicrobial activity against Gram-positive bacteria, the direct causal link between the detected actinomycin D and this bioactivity remains to be confirmed through compound isolation and structural validation. Similarly, other potential producers identified through mass spectrometry include *Streptomyces parvus* NBRC 3388 from marine sediments [18], as well as *Streptomyces* sp. RS3 and *Streptomyces* sp. RC4 isolated from marine invertebrates in West Kalimantan [9].

**Fig. 5:**
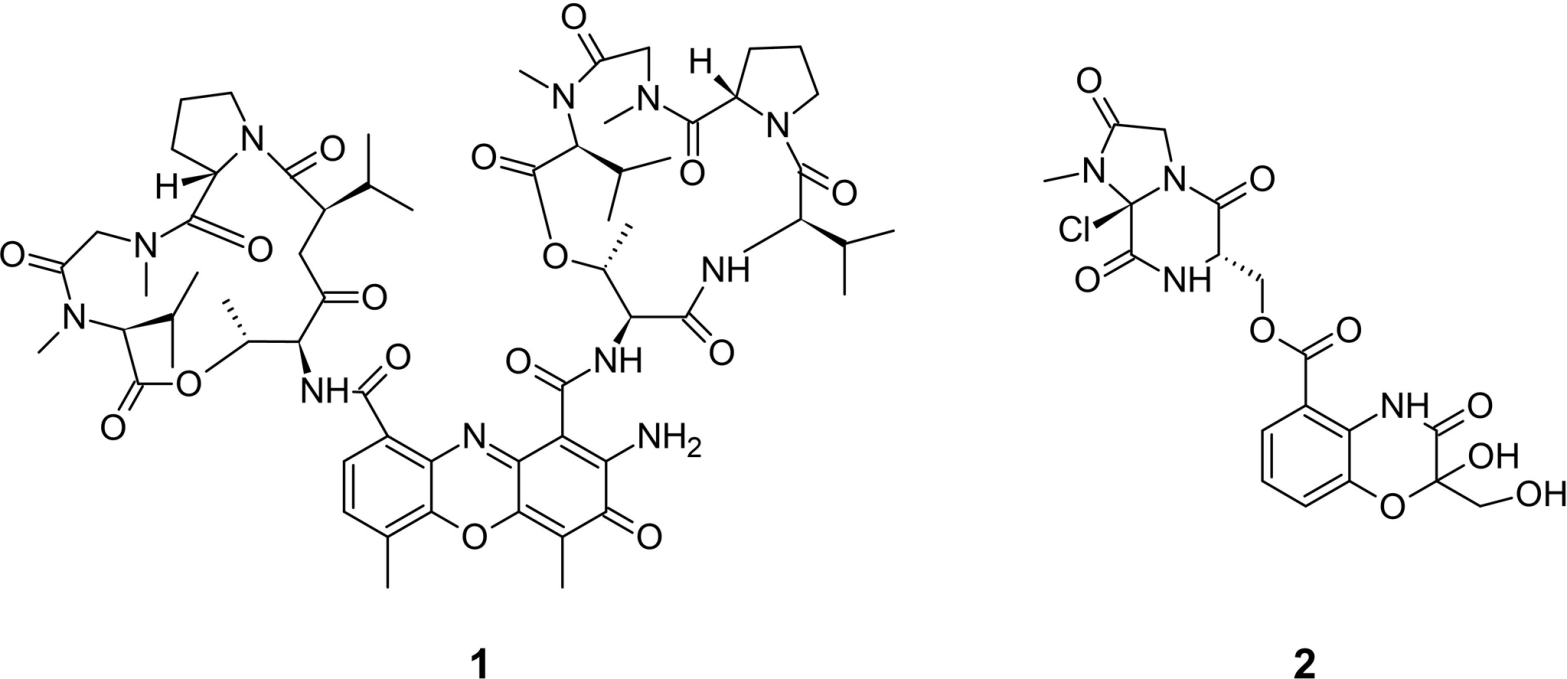
Chemical structures of cyclic peptides derived from Indonesian actinomycetes. *Tentatively identified compounds (not purified)

Ashimide B (**2**) was detected based on LC–MS analysis of the crude extract of *Streptomyces coerulescens* APM-11, isolated from soil on Muna Island, Southeast Sulawesi [34]. This cyclic peptide was first reported through heterologous expression of the *asm* gene cluster in *Streptomyces lividans* strain SBT18, and it exhibited cytotoxicity against MCF-7 cells with an IC_50_ value of 19.8 μM [111]. Although Ashimide B is a known cytotoxic agent, the biological activity of the *Streptomyces coerulescens* APM-11 crude extract in relation to this specific metabolite remains to be validated. As these identifications were largely based on chromatographic and mass spectrometric dereplication, the structures of compounds **1** and **2** in Fig. 5 are presented as proposed representations of the detected metabolite families.

### Diketopiperazines (DKPs)

Several DKPs have been reported from Indonesian actinomycetes. For instance, cyclo(Pro–Val) (**3**) was putatively identified in the ethyl acetate extracts of *Streptomyces* sp. SHP22-7 and *Streptomyces* sp. GMR22, isolated from mangrove sediment and karst soil, respectively. While these crude extracts exhibited antiplasmodial activity against *Plasmodium falciparum* FCR-3 [30], the specific contribution of compound **3** to this activity remains to be validated. Nevertheless, this compound has been reported to exhibit cytotoxicity against HCT-116 cells [112].

Another DKP, cyclo-(L-Phe–D-Pro) (**4**), was detected via LC–MS profiling in *Streptomyces* sp. RS3 and *Streptomyces* sp. RC4, which were isolated from marine invertebrates in West Kalimantan. These strains, closely related to *S. badius* and *S. microflavus*, showed antibacterial activity against *Pseudomonas aeruginosa* [9]. Although purified compound **4** has been documented to possess potent antibiotic activity against *Vibrio anguillarum* (MIC 0.10 μg/mL) [113] its presence in the Indonesian isolates was characterized based on metabolomic screening.

**Fig. 6:**
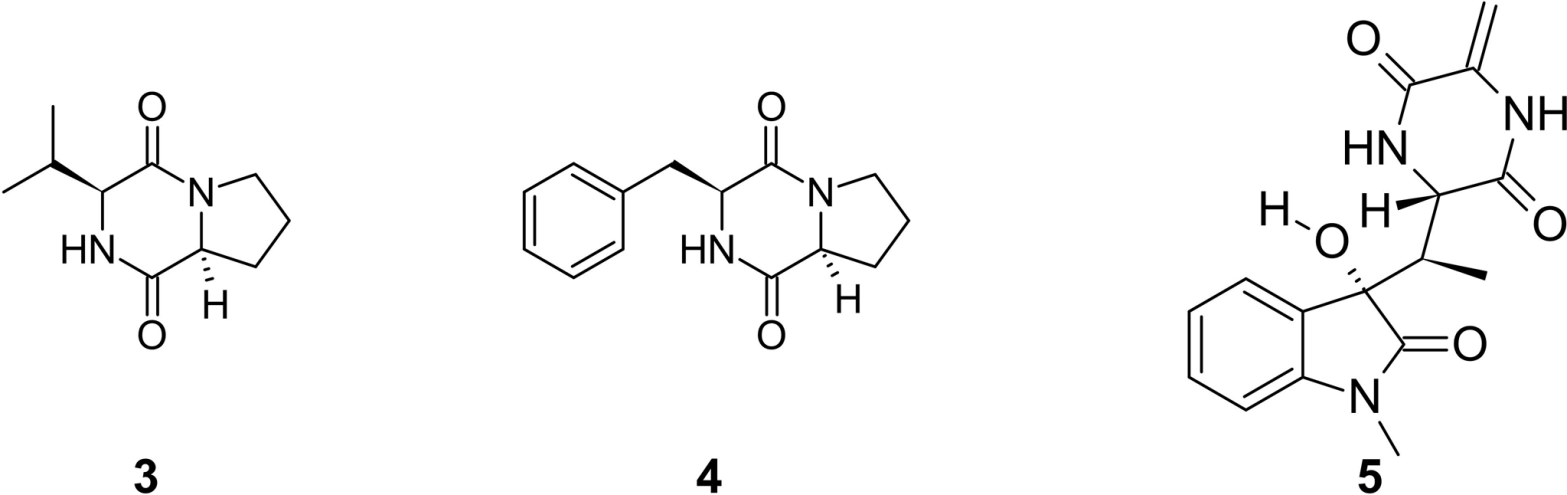
Chemical structures of the diketopiperazines (DKPs) derived from Indonesian actinomycetes. *Tentatively identified compounds (not purified)

A putative maremycin-type metabolite (**5**) was detected via LC–MS analysis in the crude extracts of *Streptomyces panayensis* APM-21 and *Streptomyces cyaneus* APM-7, both isolated from Muna Island, Southeast Sulawesi. Given that the identification was based solely on LC–MS dereplication without physical purification, the structure is presented as a representative of the maremycin family. While these crude extracts exhibited antibacterial and antibiofilm activities against various MDR clinical isolates (with MIC values ranging from 78 to 2,500 μg/mL) [34], it is important to note that the bioactivity of pure maremycins varies significantly. For instance, maremycins B and C have been reported to be largely inactive, showing only weak cytotoxicity against cell lines such as L-929, K562, and HeLa [114]. Therefore, further investigation is required to determine whether the observed antibacterial effects of the Indonesian isolates are attributable to the detected maremycin-type analog or to other coexisting metabolites in the crude extract. As these identifications were largely based on chromatographic and mass spectrometric dereplication, the structures of compounds **3**, **4**, and **5** in Fig. 6 are presented as proposed representations of the detected metabolite families.

### Polyketides

LC–MS/MS analysis of the crude extracts from three antibacterial strains, namely *Streptomyces panayensis* APM-21, *Streptomyces cyaneus* APM-7, and *Streptomyces coerulescens* APM-11, isolated from soil on Muna Island, Southeast Sulawesi, revealed the presence of several polyketide compounds. Five polyketides were putatively identified, including rancinamycin III (**6**), a putative bromoisorumbrin-type analog (**7**), paramagnetoquinone C (**8**), and enteromycin (**9**). While the crude extracts of these strains showed significant antibacterial and antibiofilm activities against MDR clinical isolates (*Escherichia coli*, *P. aeruginosa*, *Klebsiella pneumoniae*, *B. subtilis*, and *S. aureus*) [34], the individual contribution of each putatively identified polyketide to the observed bioactivity requires further validation through compound isolation.

For instance, although compound **8** was detected in active extracts, previous studies on isolated paramagnetoquinone C showed no independent activity against Gram-positive or Gram-negative bacteria [115]. Similarly, although putative bromoisorumbrin-type analog compound **7** was detected, its structural assignment remains tentative, and its reported nanomolar-range cytotoxicity is based on experimentally confirmed derivatives from other sources [116]. As the analysis was limited to LC–MS dereplication without compound purification, the chemical structure of the putative bromoisorumbrin-type compound is presented only as a representative example of the bromoisorumbrin family.

**Fig. 7:**
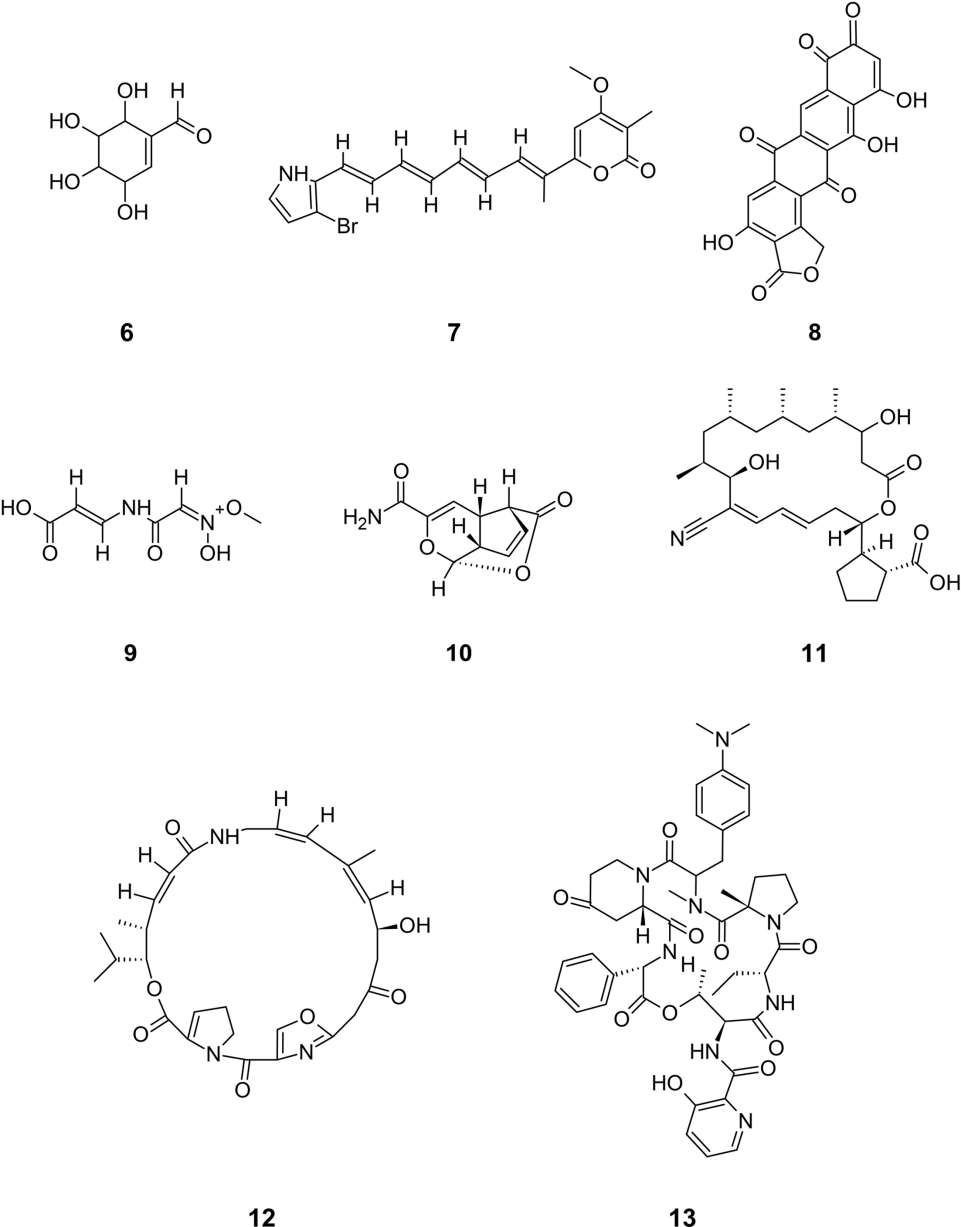
Chemical structures of polyketides derived from Indonesian actinomycetes. *Tentatively identified compounds (not purified)

In a separate study, borrelidin (**11**) was characterized using HPLC–DAD–UV–Vis analysis from the coral-associated strain *Streptomyces ardesiacus* CM11. Furthermore, the soil isolate *Streptomyces* sp. SW4 was found to possess a metabolic profile consistent with pristinamycin, containing both pristinamycin I (**12**) and II (**13**) [117]. As these identifications were largely based on chromatographic and mass spectrometric dereplication, the structures of compounds **6–13** in Fig. 7 are presented as proposed representations of the detected metabolite families.

### Alkaloids

The alkaloid compound, kuraramine (**14**) was identified in two different strains, *Streptomyces* sp. SHP22-7 and *Streptomyces* sp. BSE7-F [30]. *Streptomyces* sp. SHP22-7 was isolated from mangrove sediment on Enggano Island, North Bengkulu. Based on 16S rRNA gene sequence analysis, this strain showed 94.84% similarity to *Streptomyces rochei* NRRL B-1559^T^, which is known to produce macrocyclic antibiotics [118]. *Streptomyces* sp. BSE7-F exhibited 97.06% similarity to *Streptomyces althioticus*. Previously, compound **14** was isolated from the fresh flowers of *Sophora flavescens* [119]. Its presence in these Indonesian isolates was characterized based on metabolomic profiling.

Another alkaloid-producing strain, *Streptomyces panaciradicis* ARK13 [93], which was obtained from the soybean rhizosphere in Sukabumi, West Java, demonstrated plant growth–promoting (PGP) activity. This effect is strongly associated with its ability to produce indole-3-acetic acid (**15**) [120], a key phytohormone involved in root development and plant growth enhancement.

In contrast, the structures of nonocarbolines A–E (**16–20**) from *Nonomuraea* sp. 1808210CR [25], isolated from soil in Malang, were fully elucidated and confirmed via NMR spectroscopy. These compounds displayed weak to moderate antibacterial activity against *B. subtilis*, except for compound **15** (nonocarboline A), which was inactive. Among them, compound **17** showed strong antifungal activity against *Mucor hiemalis* (MIC 8.3 μg/mL), while compound **19** exhibited the highest cytotoxicity against human lung carcinoma A-549 cells with an IC_50_ value of 1.7 μM.

A piericidin-like alkaloid (**21**) was also detected in *Streptomyces pluripotens* CM4, an actinomycete associated with stony coral in Karimunjawa, Jepara. The compound was identified based on retention time, UV spectral characteristics, and comparison with an actinomycete metabolite database. Piericidin derivatives are well known for their broad-spectrum antimicrobial and anticancer properties [121]. Consistent with this, *Streptomyces pluripotens* CM4 demonstrated antimicrobial activity with MIC values of 15.62–31.25 μg/mL, MBC values of 15.62–62.50 μg/mL, and cytotoxic activity against P388 murine leukemia cells with an IC_50_ value of 26.54 ± 1.03 μg/mL [79].

Furthermore, two novel phenazine derivatives, compounds **22** and **23**, along with griseoluteic acid (**24**), were experimentally confirmed in *Streptomyces* sp. ICBB8198 from the Kalimantan Black Water Ecosystem [31]. Bioassays revealed that compound **22** inhibited *S. aureus* (14 mm at 24 μg/disk), while compound **24** showed potent inhibition against both *S. aureus* and *E. coli* (25 and 20 mm, respectively). Griseolutein A (**25**) and 3,6-diethyl-4-hydroxy-5-methyl-*2H*-pyran-2-one (**26**) were also identified from the same strain, with the former known for its antibacterial and antitumor activities [122, 123].

Another alkaloid compound detected was curan-17-oic acid, 2,16-didehydro-20-hydroxy-19-oxo-, methyl ester (**27**), and gentiatibetine (**28**). These metabolites were reported from *Streptomyces* sp. BSE7-F [31], isolated from mangrove sediment on Enggano Island, North Bengkulu [30]. These two compounds have been reported to exhibit antiyeast activity [124] and anticonvulsant effects [125], respectively.

**Fig. 8:**
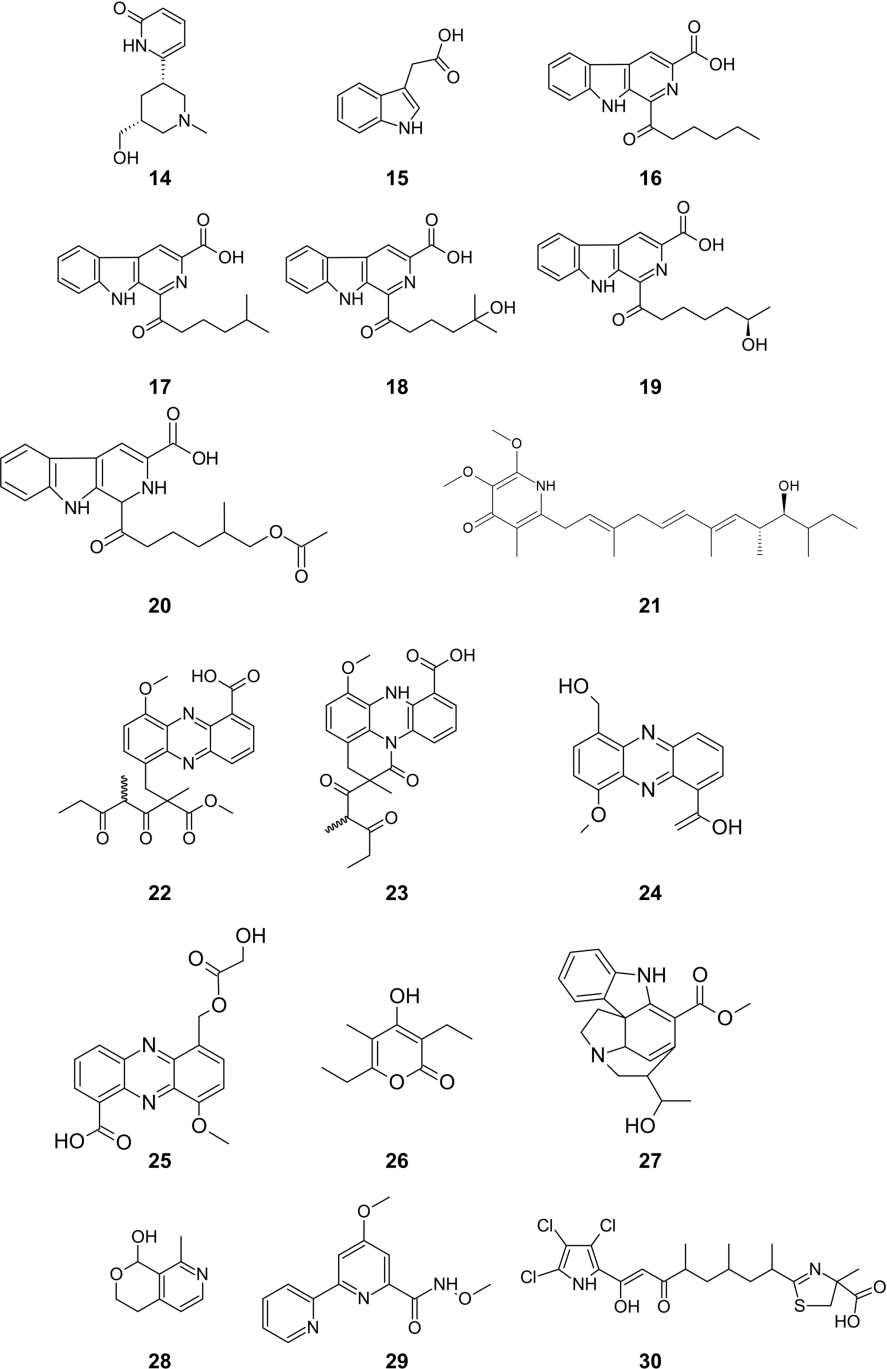
Chemical structures of alkaloids derived from Indonesian actinomycetes. *Tentatively identified compounds (not purified)

Finally, LC–MS/MS analysis of antibacterial crude extracts from *S. panayensis* APM-21 and *Streptomyces coerulescens* APM-11 indicated the presence of caerulomycin I (**29**) and thiazohalostatin (**30**). Compound **29** has been documented as a potent inhibitor of K562 cells, with an IC_50_ value of 0.37 μM [126], while compound **30** is recognized for its cytoprotective activity and inhibition of lipid peroxidation [127]. The chemical structures of compounds **14–30** are presented in Fig. 8. Within this figure, compounds **16–20** and **22–24** are depicted as confirmed structures based on full structural elucidation via NMR spectroscopy. In contrast, the structures of metabolites **14, 15, 21**, and **25–30** are provided as proposed representations, reflecting their putative identification through metabolomic profiling and database matching.

### Terpenoids

Terpenoid compounds have also been reported from Indonesian actinomycetes. A study from Enggano Island, North Bengkulu, putatively identified two terpenoid metabolites: 12-hydroxy-methyl abietate (**31**) from *Streptomyces* sp. BSE7-F and poricoic acid F (**32**) from *Streptomyces* sp. SHP22-7 [30]. While specific bioactivity data for compound **31** remain limited, related abietane diterpenoids are recognized for their potential antifungal activities [128]. In contrast, compound **32** exhibited potent antifibrotic activity and was reported to promote angiogenesis and myocardial regeneration [129, 130].

**Fig. 9:**
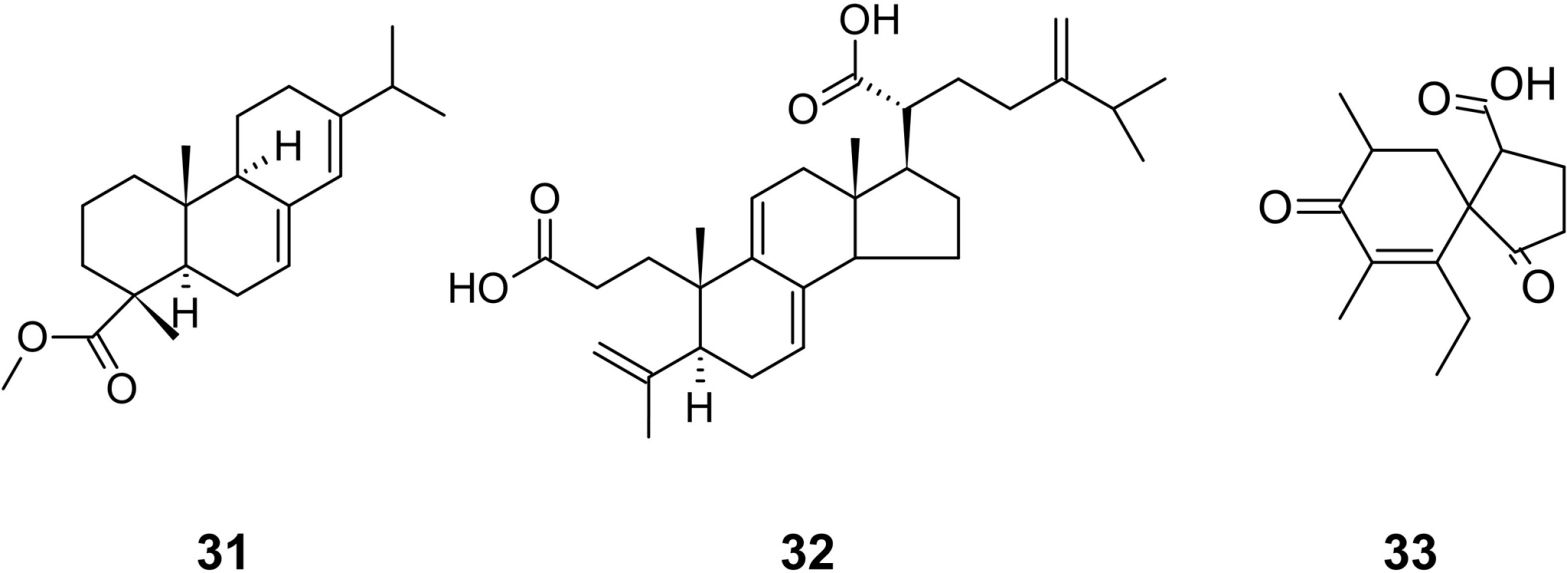
Chemical structures of terpenoids derived from Indonesian actinomycetes. *Tentatively identified compounds (not purified)

Another terpenoid compound, spirodionic acid (**33**), was detected in *Streptomyces* sp. ICBB8198 isolated from the Black Water Ecosystem, Kalimantan [31]. Studies on this compound remain limited due to its low yield [131]. As the identification of these metabolites in the Indonesian isolates was based on LC–MS/MS dereplication, the chemical structures of compounds **31**, **32**, and **33** in Fig. 9 are presented as proposed representations.

### Phenyl-Substituted Compounds

Several phenyl-substituted compounds have been detected from Indonesian actinomycetes. A phenolic compound, 2,6-*bis*(4-hydroxyphenyl)-3′,5-dimethoxy-3-hydroxybibenzyl (**34**), was putatively detected in *Streptomyces* sp. SHP22-7, isolated from North Bengkulu [30]. While research on compound **34** remains limited, moscatilin—a phenolic compound with a structurally similar scaffold—has been documented for its potential anticancer effects [132].

**Fig. 10:**
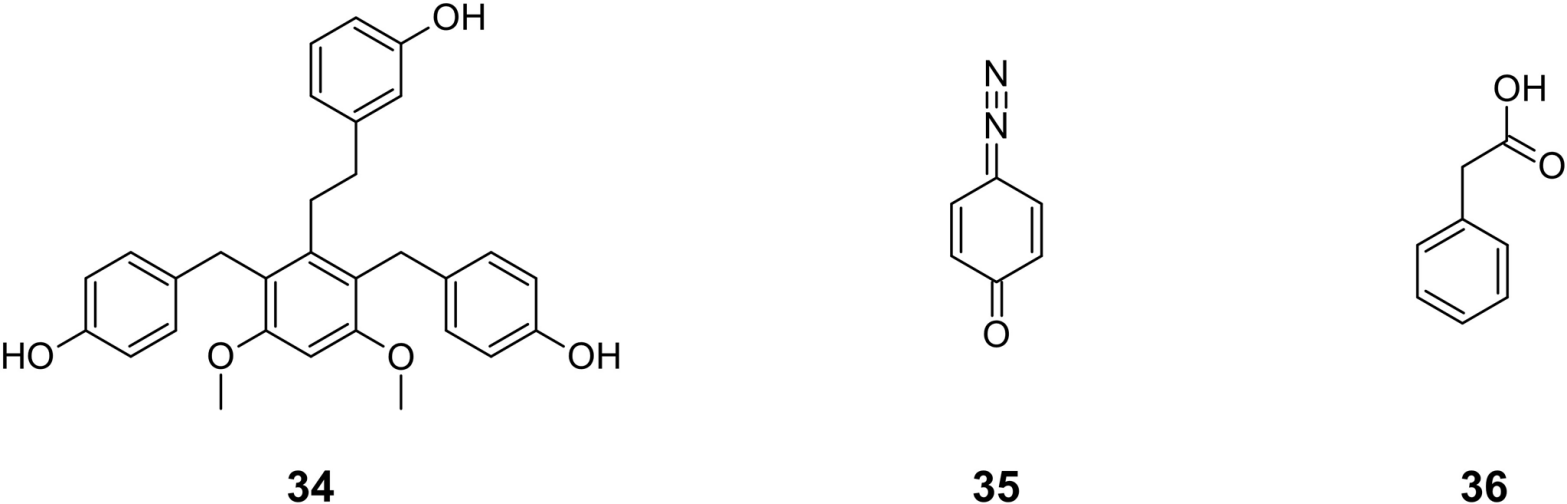
Chemical structures of phenyl-substituted compounds derived from Indonesian actinomycetes. *Tentatively identified compounds (not purified)

Furthermore, 4-hydroxybenzenediazonium hydroxide inner salt (**35**) was identified via metabolomic profiling in *Streptomyces* sp. ARJ 16, isolated from the rhizosphere soil of maize [95]. Compound **35** has been reported in the literature to exhibit antibacterial activity against *E. coli*, *P. aeruginosa*, and *Salmonella typhi* [133]. Another strain isolated from the same site, *Streptomyces* sp. ARJ 36 [95], was found to produce phenylacetic acid (**36**), a carboxylic acid derivative known for its antibacterial activity against pathogens associated with human ear infections [134]. This compound has also been reported to exhibit antimicrobial activity [135]. Given that these identifications were based on dereplication methods, the chemical structures of compounds **34**, **35**, and **36** in Fig. 10 are presented as proposed representations.

### Miscellaneous Compounds

In addition to the major compound classes, several other bioactive metabolites were detected via metabolomic screening in *Streptomyces* sp. ARJ16, *Streptomyces* sp. ARJ24, and *Streptomyces* sp. ARJ36, isolated from maize rhizosphere soil in East Nusa Tenggara. These included furan derivatives such as 2-furaldehyde (**37**), furan-2-carboxylic acid (**38**), and 5-(hydroxymethyl)furfural (**39**); a cyclopentane derivative, 1,3-cyclopentanedione (**40**); and an alcohol derivative, procurcumenol (**41**). Although *Streptomyces* sp. ARJ16 and *Streptomyces* sp. ARJ24 were both closely related to *Streptomyces* sp. TES 17 based on 16S rRNA gene analysis, their distinct secondary metabolite profiles suggest that they represent different strains [95].

Compound **37** (furfural) and compound **38** (furoic acid) have been reported to inhibit the tyrosinase enzyme and to exhibit antimicrobial effects against *Salmonella* spp. and *B. subtilis* [136]. Compound **39** is well documented for its potent antisickling activity, alongside a wide range of bioactivities, including antiallergic, antioxidant, and anti-inflammatory effects [137]. Compound **40** has been evaluated for its medicinal chemistry potential as a bioisostere of carboxylic acid moieties in drug design, interacting with biological targets such as thromboxane A_2_ receptors [138]. The activity of compound **41**, including its antioxidant properties and weak cytotoxicity, has also been widely reported [139, 140].

**Fig. 11:**
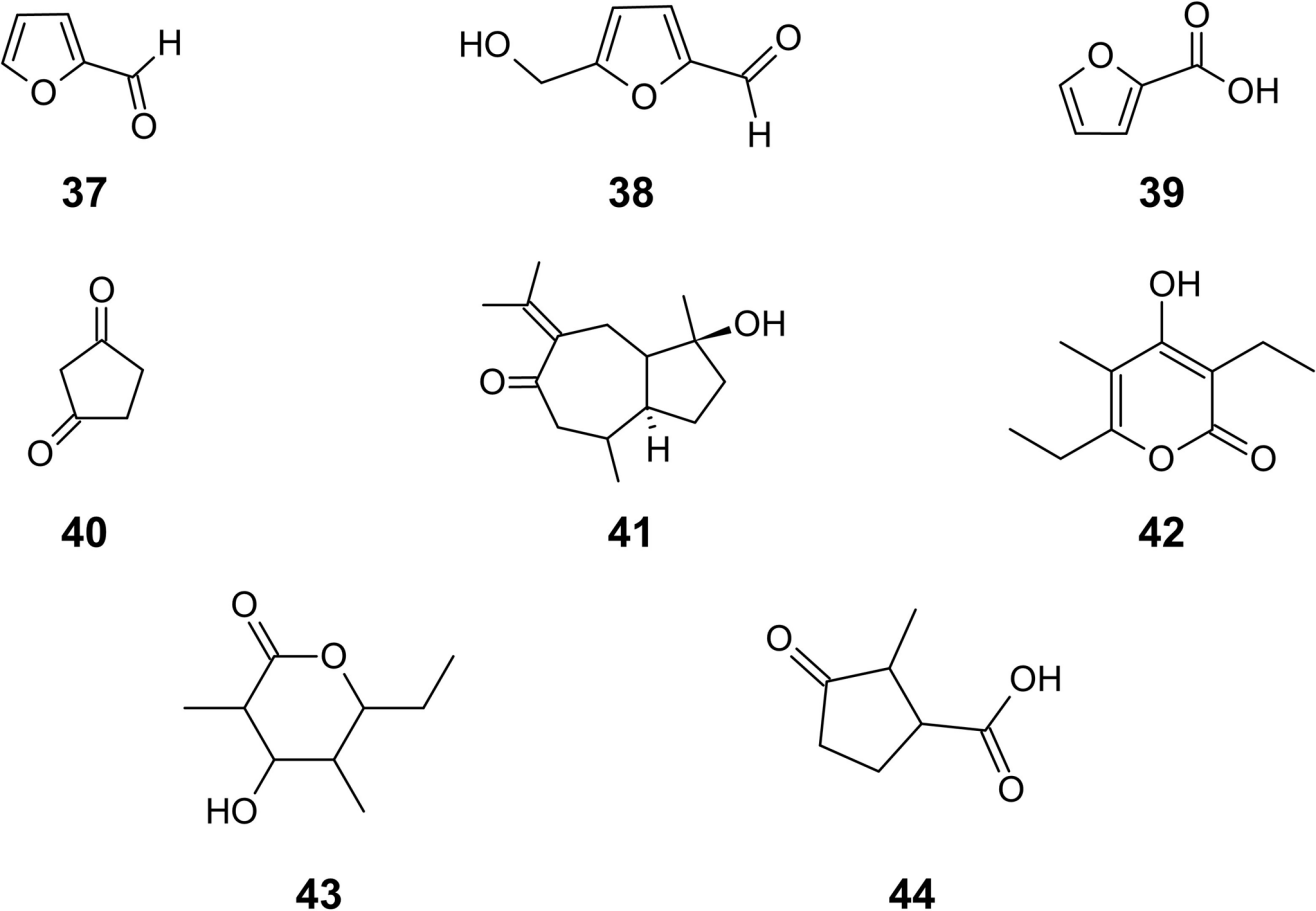
Chemical structures of miscellaneous compounds derived from Indonesian actinomycetes. *Tentatively identified compounds (not purified)

Additionally, heterocyclic compounds such as 3,6-diethyl-4-hydroxy-5-methyl-*2H*-pyran-2-one (**42**) and 6-ethyl-4-hydroxy-3,5-dimethyl-*2H*-pyran-2-one (**43**) were isolated and confirmed via NMR spectroscopy from *Streptomyces* sp. ICBB8198, isolated from the Black Water Ecosystem in Kalimantan. However, both α-pyrone metabolites were found to be inactive when evaluated individually in antibacterial assays [31]. Additionally, dihydrosarkomycin (**44**), a macrolide polyketide, was experimentally confirmed via NMR spectroscopy in the same study. Information on this compound remains limited; however, its parent compound, sarkomycin—the nonhydrogenated form—is known for its antitumor activity [141]. Unlike the putatively identified metabolites **37–41**, the structures of compounds **42–44** in Fig. 11 are presented as confirmed chemical entities.

To illustrate the diversity of reported secondary metabolites, selected findings from various Indonesian actinomycete strains are summarized in Table 8. In cases where compounds were putatively identified through LC–MS/MS dereplication, their potential biological activities were cross-referenced with previous studies to provide a comprehensive overview of the chemical landscape.

## Conclusion

The biodiversity and natural products of actinomycetes isolated from various environments in Indonesia are highlighted in this review. Diverse environmental sources of actinomycetes in Indonesia include soil, karst ecosystems, geothermal areas, mangrove ecosystems, marine organisms, and plant-associated environments. According to the literature, research on actinomycetes has predominantly focused on soil-derived isolates, often leading to the rediscovery of previously reported strains or natural products. Furthermore, studies have largely been conducted on Java Island, with limited exploration in other regions, even though these regions possess unique environmental conditions that may facilitate novel discoveries. This geographical disparity is further compounded by the fact that most previous papers failed to provide exact GPS coordinates, limiting the precise mapping and spatial evaluation of Indonesia's actinomycete diversity. The number of research teams specifically focusing on actinomycetes is also limited and primarily concentrated on Java Island. For future studies, there is a need to expand both the geographical coverage and improve the precision of locational data reporting, and increase the number of research teams investigating actinomycetes outside Java Island.

Biodiversity analysis was conducted through phylogenetic tree construction, which requires valid genetic sequencing data. However, most studies in Indonesia have relied mainly on conventional identification methods, such as morphological characterization, which are largely qualitative and often insufficient for accurate species-level identification. Moreover, in many studies involving hundreds of actinomycete isolates, DNA-based identification has been performed only on selected strains exhibiting potential bioactivity. This practice may result in the loss of biodiversity data and missed opportunities to identify novel actinomycete species.

In this review, phylogenetic trees were constructed using 152 strains with available accession numbers obtained from published research and public databases such as NCBI. Of these, 26 strains were derived from open-source submissions in NCBI (unpublished records) that are publicly accessible, while the remaining 126 strains were obtained from only 47 published journal articles. These findings suggest that molecular-level studies on actinomycetes in Indonesia remain limited. Therefore, greater emphasis on DNA sequencing and GenBank submission is strongly recommended. Incorporating comprehensive genetic data is essential not only to prevent species misidentification and redundant discoveries but also to support future research in bioactive compound discovery, phylogenetics, and biotechnological applications.

Based on the constructed phylogenetic tree, actinomycetes in Indonesia belonged to two distinct groups, *Streptomyces* and non-*Streptomyces*, with the genus *Streptomyces* representing the largest proportion. Notably, 52.3% of Indonesian actinomycetes belong to the genus *Streptomyces*, and most were isolated from soil environments. In contrast, rare actinomycetes, comprising six different clades, were predominantly isolated from alternative environments and were mainly associated with marine habitats. This analysis highlights the broad taxonomic distribution and substantial ecological potential of Indonesian actinomycetes across both terrestrial and marine ecosystems.

Various natural products have been reported from actinomycetes isolated from different environments in Indonesia. These compounds are classified into several chemical groups: cyclic peptides, DKPs, polyketides, alkaloids, terpenoids, phenyl-substituted compounds, and miscellaneous compounds. The chemical structures of these compounds are presented and their bioactivities are described as previously documented. In this review, 44 compounds were identified. However, this number remains relatively low compared to global benchmarks, where specific environments have yielded more than 500 compounds [73]. Consequently, natural product research in Indonesia remains significantly underexplored.

A critical observation from this review is that, although most strains were evaluated for antimicrobial or antifungal activities, these assays were predominantly conducted as preliminary screenings using crude extracts. Only a small fraction of these studies progressed to intensive bioassay-guided isolation and full structural characterization, for instance, through NMR spectroscopy. This reliance on putative identification through metabolomic profiling without subsequent compound purification limits definitive confirmation of novel bioactive scaffolds and hinders their progression into drug development.

Therefore, future research in Indonesia should prioritize a transition from basic molecular identification to comprehensive metabolite purification and characterization. Such efforts are essential to distinguish between known dereplicated compounds and truly novel chemical entities. By bridging the gap between metabolomic screening and structural elucidation, Indonesian research can move beyond an “underexplored” status and make a significant, validated contribution to the global natural product discovery landscape.

## Figures and Tables

**Table 1:** Literature search record.

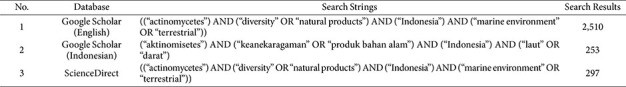

**Table 2:** Summary of actinomycetes isolated from soil in various regions of indonesia.

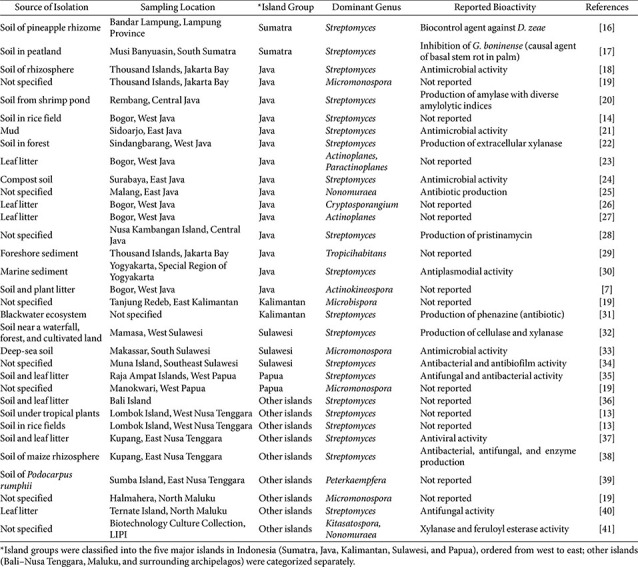

**Table 3:** Summary of actinomycetes isolated from karst ecosystems in various regions of indonesia.

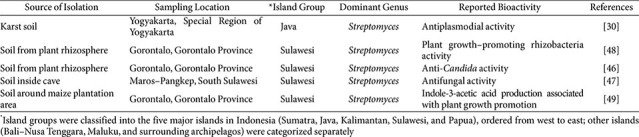

**Table 4:** Summary of actinomycetes isolated from geothermal areas in various regions of indonesia.

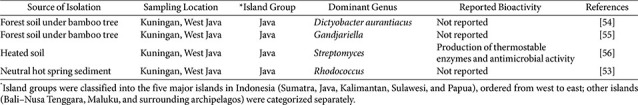

**Table 5:** Summary of actinomycetes isolated from mangrove ecosystems in various regions of indonesia.

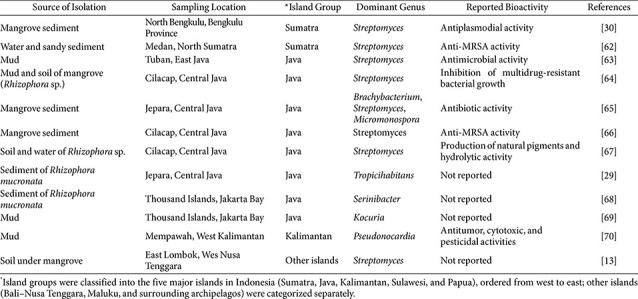

**Table 6:** Summary of actinomycetes isolated from marine organisms in various regions of indonesia.

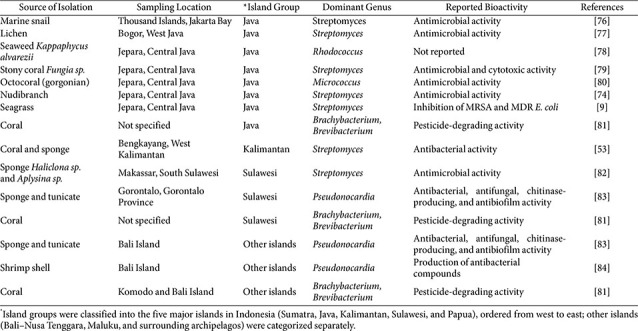

**Table 7:** Summary of actinomycetes isolated from plant-associated sources in various regions of indonesia.

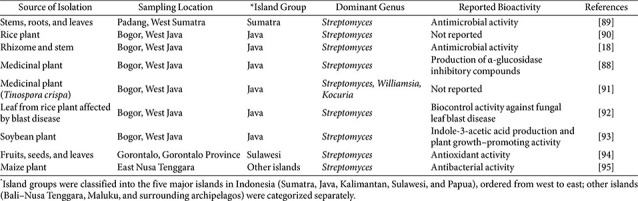

**Table 8:** Summary of reported secondary metabolites from Indonesian actinomycetes.